# *In*-*situ* high-speed X-ray imaging of piezo-driven directed energy deposition additive manufacturing

**DOI:** 10.1038/s41598-018-36678-5

**Published:** 2019-01-30

**Authors:** Sarah J. Wolff, Hao Wu, Niranjan Parab, Cang Zhao, Kornel F. Ehmann, Tao Sun, Jian Cao

**Affiliations:** 1 Department of Mechanical Engineering, Northwestern University, Evanston, IL 60208 USA; 2 School of Mechanical Engineering and Automation, Northeastern University, Shenyang, 110819 People’s Republic of China; 3 X-ray Science Division, Advanced Photon Source, Argonne National Laboratory, Lemont, IL 60439 USA

## Abstract

Powder-blown laser additive manufacturing adds flexibility, in terms of locally varying powder materials, to the ability of building components with complex geometry. Although the process is promising, porosity is common in a built component, hence decreasing fatigue life and mechanical strength. The understanding of the physical phenomena during the interaction of a laser beam and powder-blown deposition is limited and requires *in*-*situ* monitoring to capture the influences of process parameters on powder flow, absorptivity of laser energy into the substrate, melt pool dynamics and porosity formation. This study introduces a piezo-driven powder deposition system that allows for imaging of individual powder particles that flow into a scanning melt pool. Here, *in*-*situ* high-speed X-ray imaging of the powder-blown additive manufacturing process of Ti-6Al-4V powder particles is the first of its kind and reveals how laser-matter interaction influences powder flow and porosity formation.

## Introduction

Additive manufacturing (AM) provides the opportunity to investigate every aspect of process-structure-properties relationships^1–3^ and build structurally-optimized^4,5^, complex^6^, and functionally-graded components^7–9^. Rapid directional solidification^10^ in additive manufacturing provides novel means for materials design^11^ with complex geometries and functionalities^12^. The majority of experimental work in additive manufacturing focuses on the resulting structure and mechanical behavior, which are characterized *ex-situ*, or after the process is completed and the additively-built component has cooled down^13^. Isolating the influences of process parameters on microstructure has been a challenge because the thermal history of a build is sensitive to slight changes in the process^14,15^. Monitoring of advanced manufacturing processes to evaluate changes in thermal history, structure and properties is crucial for an understanding of the physical phenomena that occur during the process and for closed-loop control of built properties in AM^16–18^. In particular, thermal models of powder-blown additive manufacturing processes use simplified laser absorptivity parameters for the substrate and powders even though absorptivity changes with laser power, scan speed, powder flow rate, the laser induced vapor-plasma plume and the inert gas environment^18–22^. The predicted thermal histories and structures from such models are highly sensitive to laser energy absorptivity during the process^23–26^, underscoring the need for experiments that reveal how the substrate and powder deposition absorb the laser beam.

The powder-blown additive manufacturing process, or directed energy deposition (DED), is characterized by the interaction of a heat source with powder particles that flow from a nozzle into a melt pool^2^. DED has been growing in pervasiveness, mostly in component repair and prototyping. In comparison to powder bed systems, DED undergoes different heat transfer mechanisms with more directional solidification as building an additional layer does not depend on the time it would take to spread a new layer of un-melted particles^2^. In addition, DED has greater flexibility in that the deposited particles can be a mixture of materials that flow from multiple nozzles with the capability to build gradient material structures. Due to the complexity of deposition of powders and their interactions in DED, however, monitoring the influence of individual particles on the melt pool and the resulting build is challenging^27^. In this study, a low-cost piezo-driven powder delivery system is used to deposit individual particles as they interact with a moving laser beam as means to capture the underlying physics of laser-matter interaction during DED using high-speed X-ray imaging.

Key past work that used *in-situ* monitoring of additive manufacturing to understand the fundamental physical interactions have highlighted how unique additive manufacturing is in comparison to conventional methods such as welding and casting due to its rapid solidification. In addition to *in-situ* monitoring of thermal profiles with infrared cameras^28,29^, two-wave pyrometers^30^ and thermocouples^31^, researchers at Lawrence Livermore National Laboratory used ultra high speed imaging of laser powder bed fusion and found that vapor driven entrainment as opposed to widely believed recoil pressure led to particle spattering which could lead to defects in the component^32^. The first of its kind, *in-situ* high-speed X-ray imaging of laser-matter interaction in a powder bed system using the Advanced Photon Source at Argonne National Laboratory revealed the capability to monitor the real-time growth of the melt pool geometry and its resulting solidification rate, particle ejection speeds out of the melt pool of tens of meters per second, and key hole porosity formation within 50 *μ*s in a laser powder bed fusion system^33^. Other key studies that contribute to the time-resolved mechanisms of laser-matter interactions in a powder-bed system include ultrafast X-ray diffraction^34^, X-ray imaging and diffraction with a scanning laser^35^, and *in-situ* X-ray imaging of Marangoni convection driven porosity formation^36^.

This study uses the high-resolution, time-resolved X-ray imaging technique at the Advanced Photon Source to evaluate the interactions between the laser beam, flying particles, and the melt pool behavior during rapid solidification in DED. By expanding this advanced monitoring technique to AM processes, researchers can take advantage of the fundamental physics of particle flow, fluid flow and heat transfer to take AM into a revolutionary means for scalable operations with flexible and functional materials.

## Experimental Setup

### Overall setup and triggering sequence

**Figure 1:**
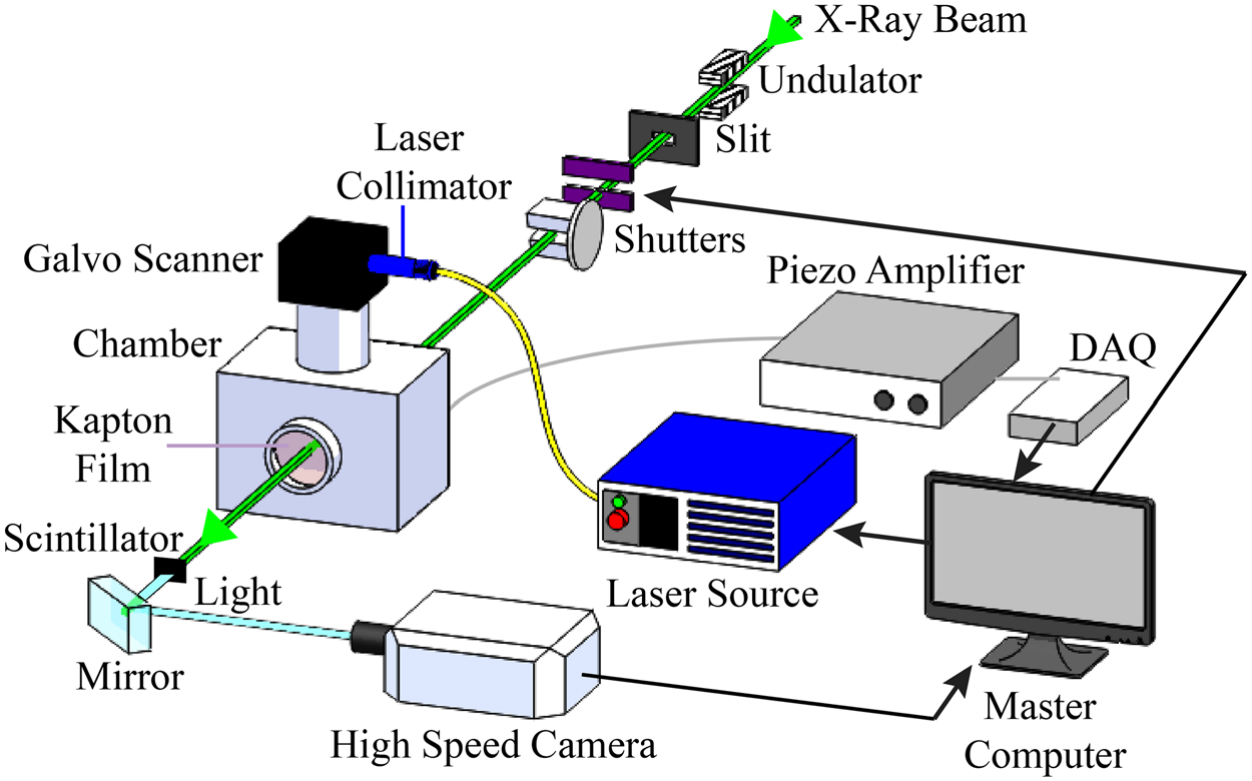
Schematic of the beamline setup with the DED process within the chamber.

The overall schematic of the experimental system is shown in Fig. 1. The piezo-driven powder delivery system and sample are housed in a sealed chamber with an argon gas environment to be described in detail below. The 500 W continuous wave Ytterbium fiber optic laser with a wavelength of 1070 nm is collimated and delivered through a galvanometer scanner that illuminates the beam onto the substrate in the chamber. The laser beam diameter is about 80 *μ*m and aligned with the deposition area of the piezo-driven powder delivery system at the top surface of 500 *μ*m thick Ti-6Al-4V Grade 5 sheet substrates.

The triggering sequence begins with the actuation of the piezo element in the powder delivery system. The signal from the piezo element switches the laser on. The “on” signal from the laser begins the laser scan by moving the galvo scanner mirrors. When the galvo mirrors are at a certain position that aligns the laser beam with the piezo-driven powder delivery system, the X-ray shutter open to allow the X-ray beam traverse into the chamber.

An undulator, a component that consists of a periodic structure of dipole magnets, is inserted into an X-ray source to oscillate electrons and therefore, radiate energy. The gap width of the undulator components determines the energy flux of the X-ray beam. The undulator gap sets the harmonic energy of the beam to 24.4 keV and a corresponding wavelength of 0.508 Angstroms. A pair of slits defines the size of the X-ray beam. The slits provide a 2 by 2 mm field of view, and fast and slow shutters dictate the time intervals for the beam to pass through, as seen in Fig. 1.

The X-ray beam traverses through the observation windows of the chamber covered with Kapton film, which is larger than the 2 × 2 mm field of view. The X-ray beam penetrates through the top of the substrate to monitor the scan-build plane of the process where the laser-matter interactions occur. After the X-ray traverses through the region of interest, the scintillator outside of the chamber converts the beam into visible light, which triggers detection by the high-speed camera system.

After the X-ray beam traverses the material of interest, a scintillator converts the X-ray beam to visible light. A 45° mirror reflects the visible light into the high-speed camera, which serves as an imaging detector. The resolution of the resulting images is 1024 by 1024 pixels with a pixel size of 1.97 *μ*m. The exposure time for each image is 500 ns with a frame rate of 20 kHz.

### Piezo-driven powder delivery system

Control of powder delivery in additive manufacturing processes is crucial as any change in mass flow influences the thermal gradient and geometry of the resulting melt pool, and therefore, the mechanical behavior of the built component^37^. Powder delivery in laser-based processes requires controlled mass flow into a melt pool area that is dependent on the laser beam diameter. Most powder delivery systems provide a stream of inert gas to carry powders onto the substrate with a Gaussian mass density profile, with the most effective builds occurring at the intersection of the foci of both the laser and powder.

**Figure 2:**
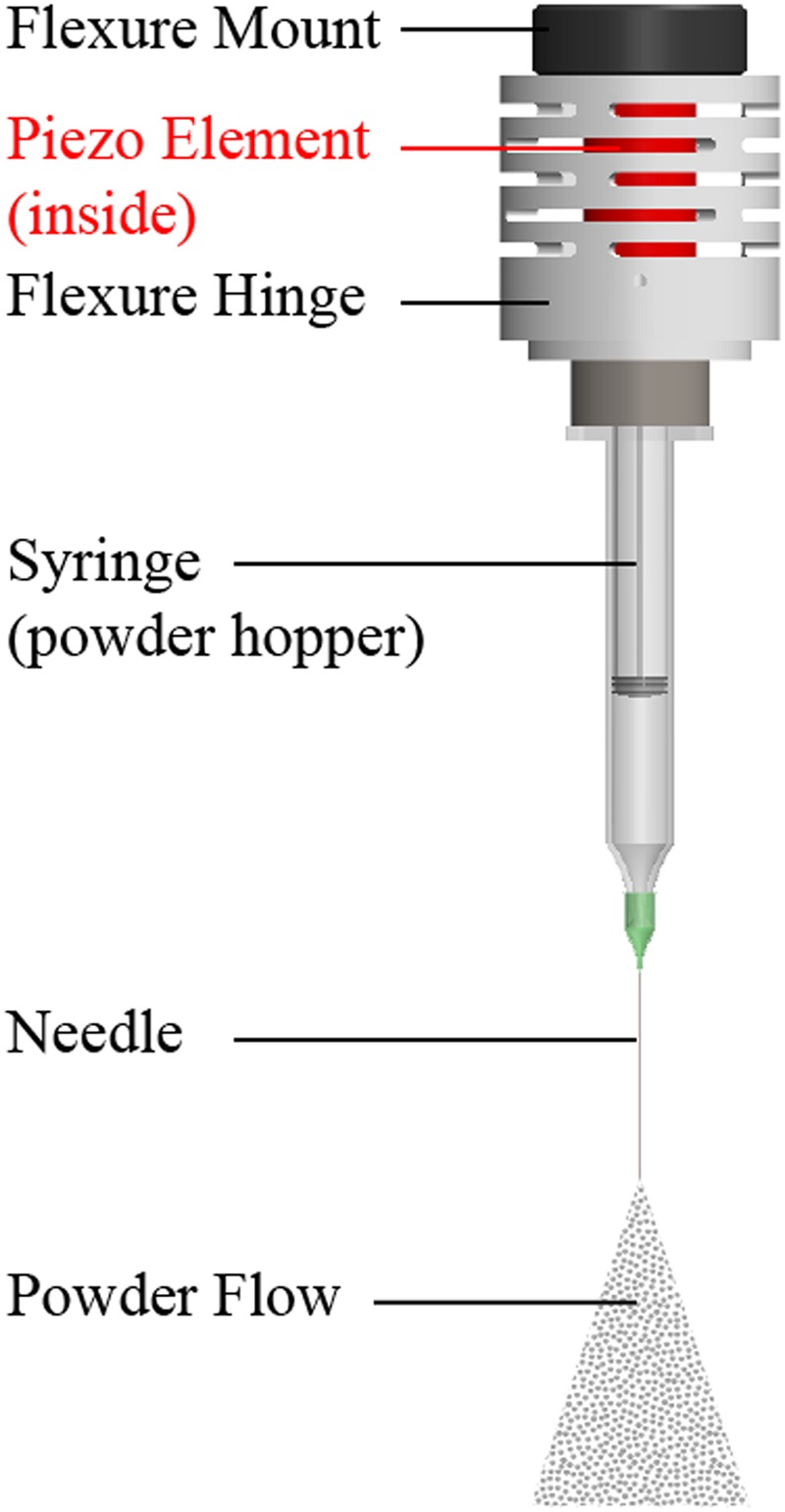
The vibration assisted powder delivery system consists of a vibration setup, piezo, syringe, and needle.

**Figure 3:**
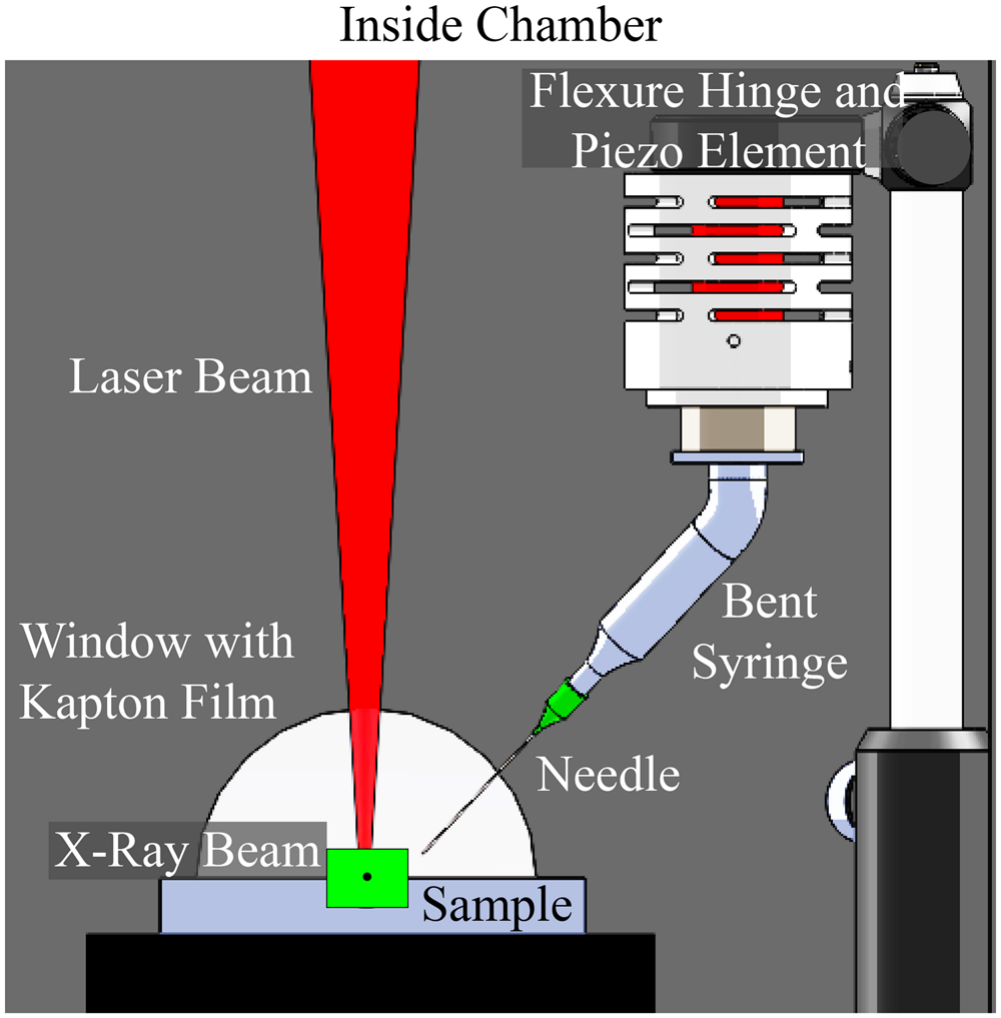
Schematic of the piezo-driven powder delivery system inside the chamber.

To better study the complex interaction between the laser beam and individual particles, a piezo-driven vibration-assisted powder delivery system is presented in this study, as seen in Fig. 2. A high-voltage amplifier controls the piezo element and provides a vertical vibration to induce a gravitational flow of powders out of the syringe-needle setup. The syringe serves as a powder hopper and the needle functions as a nozzle. The selected needle size is dependent on the surface properties, flowability, and size distribution of powder particles. In the experiments in this work, the syringe was bent at 45° relative to the laser beam path so that particles flow onto the substrate where the laser and X-ray beams align, as seen in the close-up schematic in Fig. 3, which show a view of about 25 by 25 mm within the chamber. The field of view of the images is represented by the size of the X-ray beam, which is 2 by 2 mm.

### Processing parameters

The powder mass flow rate can be controlled by modifying the vibration frequency and amplitude. Of the parameters, the most dominant factors that influence the powder mass flow rate are the vibration amplitude and frequency. Control of powder flow into a localized area presents a compact, low-cost, and efficient mechanism for powder delivery into a melt pool for imaging AM processes. The piezo-driven powder delivery system parameters used in this work include a voltage input of 400 V, a duty cycle of 10% and a range of 40 to 100 square wave pulses for each experiment. The powders used are reused Ti-6Al-4V particles of 45 to 106 *μ*m in diameter.

The DED process in this study uses a mass flow rate from 5 mg/s to 50 mg/s without inert carrier or shield gas, and therefore, the resulting flow velocity of the powder particles range from 0.1 to 0.5 m/s. In comparison, industrial DED applications use powder mass flow rates of about 50 to 500 mg/s with carrier and shield gas^2^, which leads to particle flow velocities of 1 to 5 m/s. The higher powder mass flow rates in industrial DED require greater energy density over time to melt the deposited particles, and therefore require a relatively slow moving laser beam to substantially fuse large amounts of in-flight material. Typical laser scan speeds in industrial DED are about 5 to 20 mm/s^2^. The laser scan speeds in this work with small-scale DED processing range from 50 to 500 mm/s, or about an order of magnitude greater than those in industrial processing. The greater scan speeds in this work are selected to scale the process in order to scale to the low-dosage of powder mass flow rates, achieve a melt pool shape representative of the DED process, allow for high-speed X-ray imaging to capture a moving melt pool boundary, and to illustrate the capability of high-speed, scaled-down DED processing.

Industrial DED systems use a focused laser beam diameter of 500 *μ*m to 5 mm^2^ and design their delivered powder flow so that mass flows into a spatial distribution equivalent to the laser beam diameter to ensure powders fuse into the melt pool. Monitoring large-scale, high-speed powder flow coupled with a large laser beam is difficult as the field of view is compromised at greater frame rates. Low mass flow rates coupled with a laser beam diameter of 80 *μ*m in this work allow for the isolation and imaging of individual particle flow relative to the melt pool. Overall, the selected processing parameters in this work enable optimal high-speed, high-resolution X-ray imaging that penetrates the material and shows a representative melt pool observed in additive manufacturing.

## Results and Discussion

The following results discuss the behavior of powder particle flow from the piezo-driven device and how individual particles flow above the substrate surface. Since the particles are gravitationally fed into the melt pool, there are no external factors to drive particle deposition, such as inert carrier gas. The section following the powder particle discussion elaborates on Marangoni convection of the melt pool and its similarities with particle entrainment during powder-bed processes. The uniqueness of this study is in particle deposition behavior. Cooling behavior can be attributed to process parameters, powder flow, porosity formation, porosity evolution, and mass change into or out of the melt pool.

### Piezo-driven powder delivery flow characteristics

In-flight particle velocities are determined by tracking the trajectory of the center of particles among each 2D frame using the MtrackJ ImageJ plugin. This calculates the projected velocity of the particle and hence provides the lower bound for particle scattering velocity. By considering the pixel size of 1.97 *μ*m and a frame rate of 20 kHz, particle velocities in a 2D plane can be estimated. The gravitational flow of the powders result in velocities of powders toward the melt pool of about 200 to 600 mm/s, allowing for the laser-induced vapor-plasma plume to scatter and vaporize particles. The hot laser-induced vapor-plasma plume introduces a pressure force that scatters most particles that flow from the piezo-driven needle, causing particles to dwell in the chamber atmosphere. A velocity of about 1 m/s is required for a particle from more than 200 *μ*m above the substrate surface to enter the melt pool^37^, prohibiting the majority of the piezo-driven and gravity-fed particles to enter the melt pool. However, there are a few situations where gravity-fed particles enter the melt pool, as described in the next sections.

### Marangoni convection behavior of the melt pool

**Figure 4:**
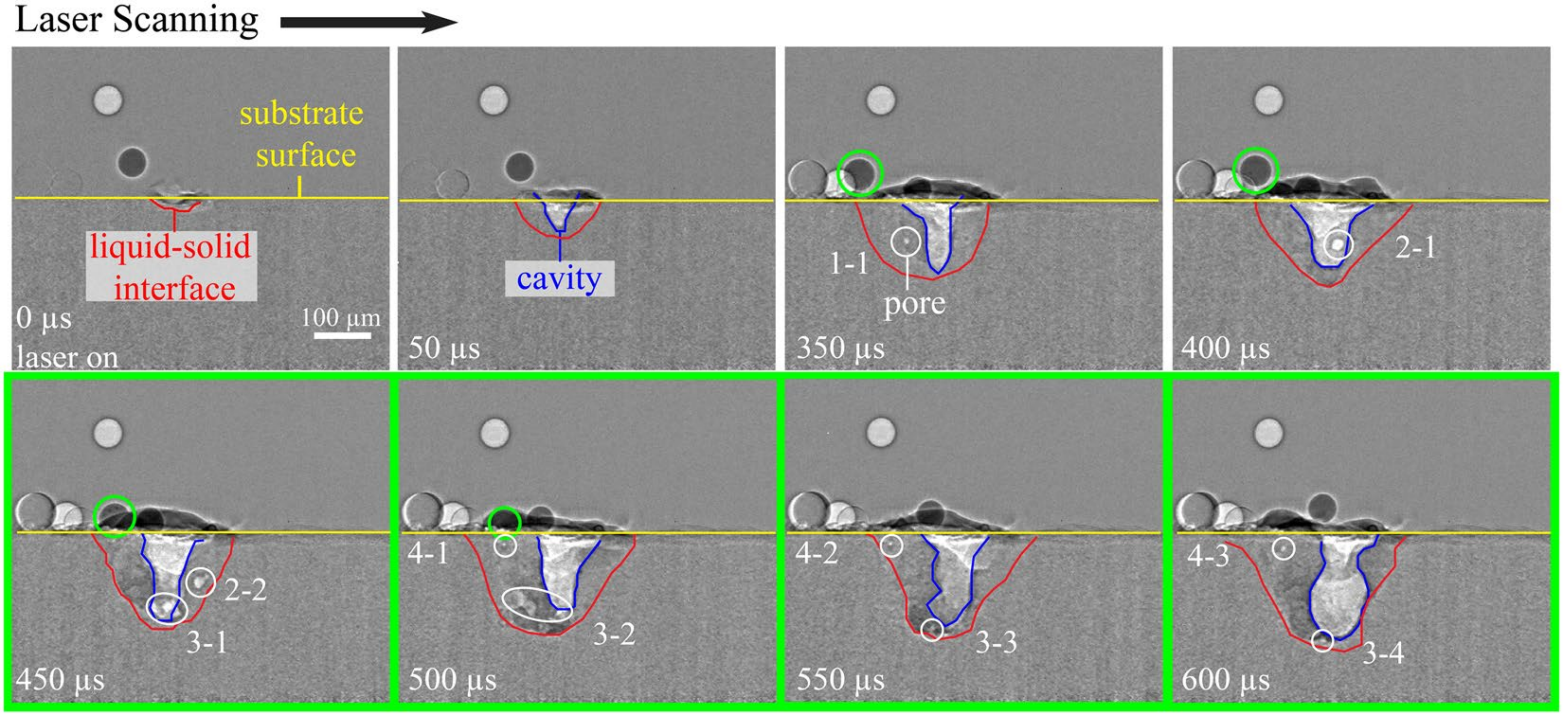
Cavity, melt pool and porosity evolution as mass is added due to surface tension in an experiment with a laser power of 250 W and a scan speed of 100 mm/s. All frames are processed images.

The interaction of the laser beam and substrate creates a melt pool with Marangoni convection that entrains particles into and out of the melt pool, behaving similarly to laser-matter interactions in powder-bed systems. For example, the introduction of cold particles into a stirring melt pool can lead to instability and growth of the melt pool. The surface tension of the melt pool introduces a Marangoni flow that entrains nearby flowing or stationary particles with low vertical momentum relative to the substrate surface. Figure 4 shows a keyhole mode experiment with a laser power of 250 W and a laser scan speed of 100 mm/s and the influence of mass addition on the porosity and cavity geometry. Keyhole mode occurs when the laser’s power density onto the substrate is great enough to create a cavity into the substrate that is filled with ionized vapor and ambient gas^38^.

The frames in Fig. 4 are processed images where the raw data is divided by the initial frame according to the image analysis procedure used in previous X-ray imaging^33^. High intensity, or light, pixels that represent particles above the substrate surface are artifacts from image processing, where a particle was in place in the initial frame. Low intensity particles above the substrate surface show a moving particle that is actually present in the frame and not an artifact. The intensity contrasts in the images around the melt pool reveal the phase state of the Ti-6Al-4V in the experiment. These contrasts depend on the density of the material at different phases. The melt pool above the substrate surface is represented by dark intensity pixels, representing the contrast of the liquid melt pool against the low density, lighter intensity ambient air pixels.

Below the substrate surface, the intensity profiles of each horizontal line of pixels are determined^33^. The liquid-solid interface is determined by identifying pixel intensities with values equivalent to those in the surrounding substrate material. The liquid melt pool has slightly lighter intensity pixels than the surrounding, slightly denser solid material, with the red outlines in Fig. 4 revealing the liquid-solid interface of the melt pool. The local peaks in the second derivative of the pixel intensity profile in each line highlights the boundaries of the gas-liquid interface, or the cavity. The blue outlines the cavity, where the light intensity pixels represent the vapor phase of Ti-6Al-4V. When porosity formation occurs, light intensity pixels that represent a vapor phase of pore appear in the frame below the substrate surface and within the liquid-solid interface.

The frames from 450 to 600 *μ*s in Fig. 4 are outlined in green to indicate when the particle enters and stirs within the melt pool. The geometry of the cavity fluctuates when a particle enters the melt pool. The vapor-plasma plume entrains the particle into a trajectory that follows the laser scan direction until the particle makes contact with the surface of the melt pool. At that point, as seen at 450 *μ*s, surface tension entrains the particle into the melt pool due to the particle’s low vertical momentum, or proximity to the surface.

#### Porosity evolution

In Fig. 4, porosity formation is tracked by an identifier, where the first number is a unique ID of a pore in chronological order and the second number is the lifetime of the pore in terms of number of frames. For example, the label “1-1” at 350 *μ*s is the first pore to appear during the process and 350 *μ*s is the first frame for that pore to appear. The formation of pore “1-1” is due to entrapped gas from the argon gas environment or the metal vapor in the cavity. By 400 *μ*s, pore “1” has dissolved within the melt pool, indicating a lifetime of less than 100 *μ*s. Dissolution of this pore is either due to the buoyancy force moving the pore to the surface of the melt pool, the cavity increasing in width and engulfing the pore, or the surrounding heated melt pool dissolving the pore due to increased gas solubility with a rise in temperature. Pore “2-1” is the second pore to appear during the process and 400 *μ*s is the first frame for that pore to appear. By 500 *μ*s, “2” dissolves into the cavity, resulting in the unique second pore present for two frames during the process. Because a span of 50 *μ*s passes between each frame, the lifetime of “2” can be between 50 and 150 *μ*s. Pore “2” traverses outward due to Marangoni flow and decreases in size from 325 to 312 *μ*m^2^ until it dissolves into the melt pool.

Figure 4 shows that as particle mass enters the melt pool, there is laser attenuation into the melt pool. Laser attenuation leads to a decrease in laser-induced cavity depth, porosity formation below the cavity, and overall fluctuations in melt pool geometry. As a particle melts into the melt pool, the cavity and melt pool regain their stable geometries. The melt pool either engulfs the porosity below the cavity or porosity stirs within the melt pool and dissolves into the surrounding molten Ti-6Al-4V material. Pore “3” forms as entrapped gas at the bottom of the melt pool that breaks up into a few smaller pores, as shown in “3-2” in Fig. 4. The large pore “3-1” disperses and decreases in size from 490 to 47 *μ*m^2^ at 600 *μ*s until it dissolves into the melt pool or is engulfed by the cavity before 650 *μ*s. Pore “4” forms as a particle enters the melt pool, pointing to either entrapped gas or surface defects of the Ti-6Al-4V particle causing the small pore. Pore “4” remains stationary due to its proximity to the liquid-solid interface, where the influence of Marangoni convection is minimal. Pore “4” maintains its size of about 25 *μ*m^2^ until it slightly increases in size at 600 *μ*s and then dissolves before 650 *μ*s due to increased temperature in the melt pool, which decreases the surface tension of the pore surface and increases the solubility of the entrapped gas.

**Figure 5:**
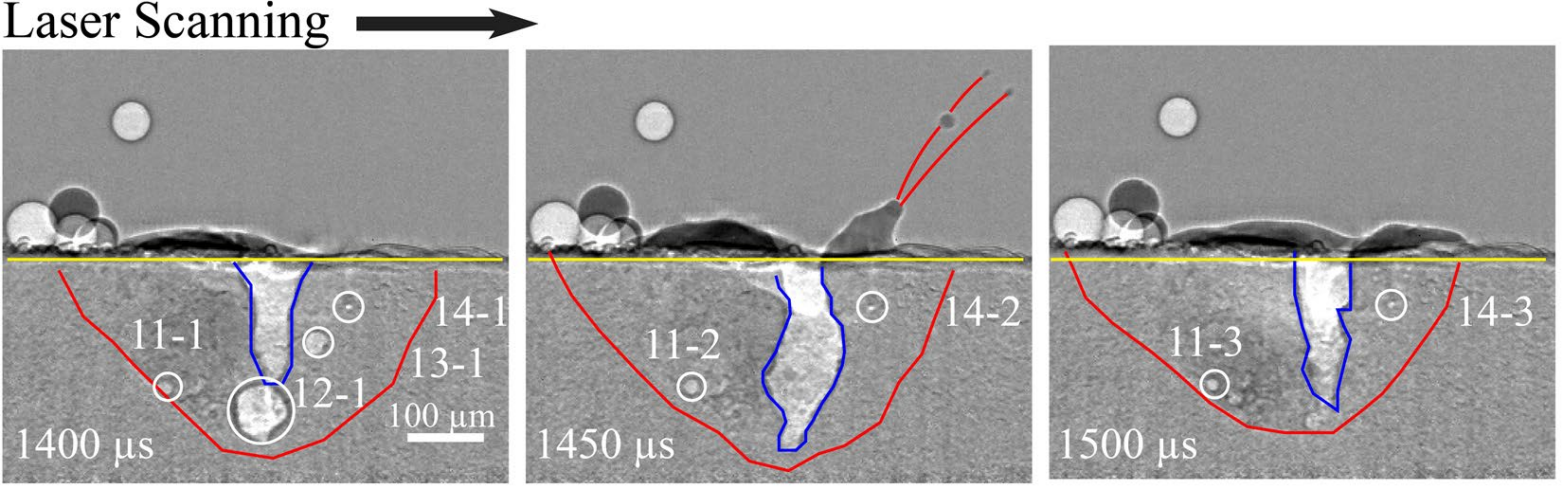
Cavity, melt pool and porosity evolution as mass is subtracted due to spattering in an experiment with a laser power of 250 W and a scan speed of 100 mm/s. All frames are processed images.

Figure 5 shows porosity formation later in the same experimental trial as Fig. 4. Porosity appears at 1400 *μ*s, in the frame before spattering occurs. Pores “11”, “13”, and “14” appear as entrapped gas porosity whereas “12” is a keyhole pore due to the collapse of the cavity when rapid shrinkage at the bottom of the melt pool occurs. The rapid shrinkage could be a result of an in-flight particle attenuating the laser or instability of the melt pool. During spattering at 1450 *μ*s, the moving cavity engulfs pores “12-1” and “13-1”, enlarging the width and depth of the cavity. The sizes of pores “11” and “14” increase during spattering at 1450 *μ*s, possibly due to both pores rising a few *μ*m toward the substrate surface, reducing the pressure of the surrounding liquid metal exerted onto the pores. Pore “11” increases in size from 217 to 271 *μ*m^2^ and pore “14” increases from 57 to 72 *μ*m^2^. After spattering at 1500 *μ*s, the melt pool shrinks in size, pore “11” breaks up into three smaller pores and pore “14” decreases in size to 42 *μ*m^2^, indicating cooling of the smaller melt pool. Though pores “11” and “14” shrink in size, they do not dissolve into the melt pool or become engulfed by the cavity until about 200 to 300 *μ*s after spattering.

Porosity evolution is tracked in the keyhole mode trials that use the same process parameters as those used in Figs 4 and 5. Over a time period of 3 ms, an average of 14 instances of porosity formation occur. Of those 14 instances of porosity formation, an average of 19.4% of the formed pores remain after the laser turns off. About half of the remaining pores undergo growth with subsequent shrinkage. Of the 80.6% formed pores that disappear during the process, 65.6% disappear by a moving cavity that engulfs the pore, 22.1% shrink and dissipate into the melt pool, and 14.4% rise to the substrate surface and dissipate. The pores that tend to shrink, dissipate and rise to the surface are less than 10 *μ*m in diameter, whereas pores that are engulfed by the cavity or remain are larger. In contrast, conduction mode experiments have an average of 4 pores that form over 3 ms, with all of the pores less than 10 *μ*m in diameter. After the laser runs off, 14.3% of the pores remain. Of those that disappear during the process, 75.0% shrink and dissipate into the melt pool and 25.0% rise to the substrate surface.

#### Spattering

Spattering arises from large pressure gradients, or recoil pressure, induced by the vapor-plasma plume and aids in the stability of the melt pool during the process. Spattering leads to the ejection of hot or molten particles from the melt pool that could deposit on other area of the build, potentially causing surface defects or increased roughness. An example of the mass subtraction via melt pool ejection is presented in Fig. 5, which is during the same experiment as shown in Fig. 4 but 600 *μ*s after the particle enters the melt pool. Figure 5 also shows the processed images to reveal the change in phase contrast during the process. As seen in Fig. 5 at 1450 *μ*s, three hot particles about 10 *μ*m in diameter or smaller eject from a volatile melt pool to the right of the cavity. The direction of the particle ejection is in the same direction of the laser scan with speeds of up to tens of m/s. During ejection, the recoil pressure from the vapor-plasma plume arising from the cavity causes the instability of the cavity geometry, increases the cavity depth, and causes spattering of hot particles. Afterwards at 1500 *μ*s, the cavity and melt pool geometry regain stability, although some porosity from 100 *μ*s prior remain in the melt pool.

Laser power, scan speed, the amount of in-flight powder particles, and the number of passes influence the spattered particle size, ejection speed, and the amount of spattering that occurs. Between the conduction and keyhole mode experiments, spattering occurs in 25% of the conduction mode experiments and 93% of the keyhole mode experiments. With an increased scan speed that designates an experiment as conduction mode, the melt pool is stable enough to resist spattering. In the 25% of the cases of spattering in conduction mode, small particles between 5 and 10 *μ*m in diameter eject at about 5 m/s.

During keyhole mode two-pass experiments, the underlying substrate retains more heat during the second pass, causing a more volatile melt pool with more spattering. In addition, an increase of temperature in the substrate reduces the surface tension at the surface of the melt pool, requiring less energy for spattered particles to escape^32^. In the 150 W keyhole mode during the first pass, an average of two particles eject out of the melt pool. The ejected particles average in size of about 20 *μ*m in diameter and eject with speeds of 1 to 8 m/s. During the second pass, an average of three particles eject out of the melt pool, with a smaller average diameter of 16 *μ*m. Ejection speeds vary from 0.5 m/s to 10 m/s. The variation in ejection speeds reflects the distribution of velocity in the induced vapor-plasma plume from the cavity. Typically smaller particles eject in the path close to center of the vapor-plasma plume at greater speeds. When the laser power increases, destabilizing the melt pool and reducing surface tension, spattering is more frequent. In the 250 W keyhole mode, the melt pool is larger and more volatile, causing more spattering. Table 1 compares the number of particles and their sizes between the 150 and 250 W laser power. The observed values aligns with observations in laser welding studies where increased laser power, or localized energy density, can increase the spattered particle size and size distribution^39^.

**Table 1:** Comparison of particle spattering between 150 and 250 W keyhole mode experiments.

2*Value	150 W		250 W	
	First pass	Second pass	First pass	Second pass
Number of ejected particles	2	3	5	7
Average diameter	20 ± 14 *μ*m	16 ± 8 *μ*m	24 ± 13 *μ*m	24 ± 21 *μ*m
Ejection speed	1 to 8 m/s	0.5 to 10 m/s	4 to 10 m/s	0.1 to 6 m/s

The number of in-flight powders in the path of the laser beam influences laser attenuation, and therefore increases the volatility of the melt pool. Increased spattering coincides with an increase of particles entering the melt pool. For all of the experiments, an average of 0.20 mg spatters for every 1 mg of particles enter the melt pool, which can be determined by simplifying the geometry all in-flight particles as spheres and measuring their diameters. When the laser power is 150 W, an average of 0.08 mg spatters for every 1 mg of mass addition and when the power is 250 W, an average of 0.33 mg spatters for every 1 mg of mass addition. This indicates that increasing both laser power, or increasing the localized energy density into the melt pool, and the number of particles that enter the melt pool can increase recoil pressure-driven spattering.

### Deposited particle behavior relative to the melt pool

DED introduces complexity to laser-matter interactions in that the amount of laser energy absorbed by either particles or the substrate fluctuates with particle flow behavior. This section highlights the uniqueness of DED phenomena, including how laser-induced vapor-plasma plume scatters mid-air individual particles more than 1 mm above the substrate surface, interactions among particles, vapor-induced pressure gradients, and a combination of both interactions with other particles and pressure gradients that can propel particles into the melt pool.

#### Particle scattering

When a laser illuminates a substrate surface with high power density, material vaporizes, forming a cavity that penetrates below the substrate surface. Process parameters influence the pressure and velocity of an expanding vapor-plasma plume, which scatter particles away from the melt pool and scatter. The scattering of particles exactly reflects the laser-induced vapor-plasma plume at the melt pool. The vapor-plasma plume reaches and pushes particles more than 1 mm above the substrate surface, resulting in particle scattering velocities between 1 and 10 m/s, depending on the size of the particle and its proximity to the melt pool. Smaller particles closer to the melt pool scatter with the greatest velocities, with accelerations up to 40 m/s^2^. Particle scattering also leads to laser attenuation, or the obstruction and scattering of the laser beam. This results in increased melt pool volatility and change in the aspect ratio of the cavity. Change in mass addition and laser scattering in DED does not allow the melt pool to arrive to equilibrium that a powder bed system would allow. In an inert environment, laser energy also ionizes the surrounding argon gas, causing a plasma plume that coincides with the metal vapor that jets from the substrate surface.

An in-flight velocity of about 1 m/s toward the melt pool is required for a particle from more than 200 *μ*m above the substrate surface to enter the melt pool^37^. Because the vapor-plasma plume pushes the particles away from the melt pool, the velocities of most of the piezo-driven and gravity-fed particles are not enough for the particles to enter the melt pool. This velocity threshold has been demonstrated in a numerical simulation of the DED process that captured the heat transfer of the laser-matter interactions during powder flow^37^. The simulated forces and velocities of the vapor from the cavity during the laser scan were plotted and could only be penetrated with a high enough particle velocity.

#### Particle interactions above the melt pool

**Figure 6:**
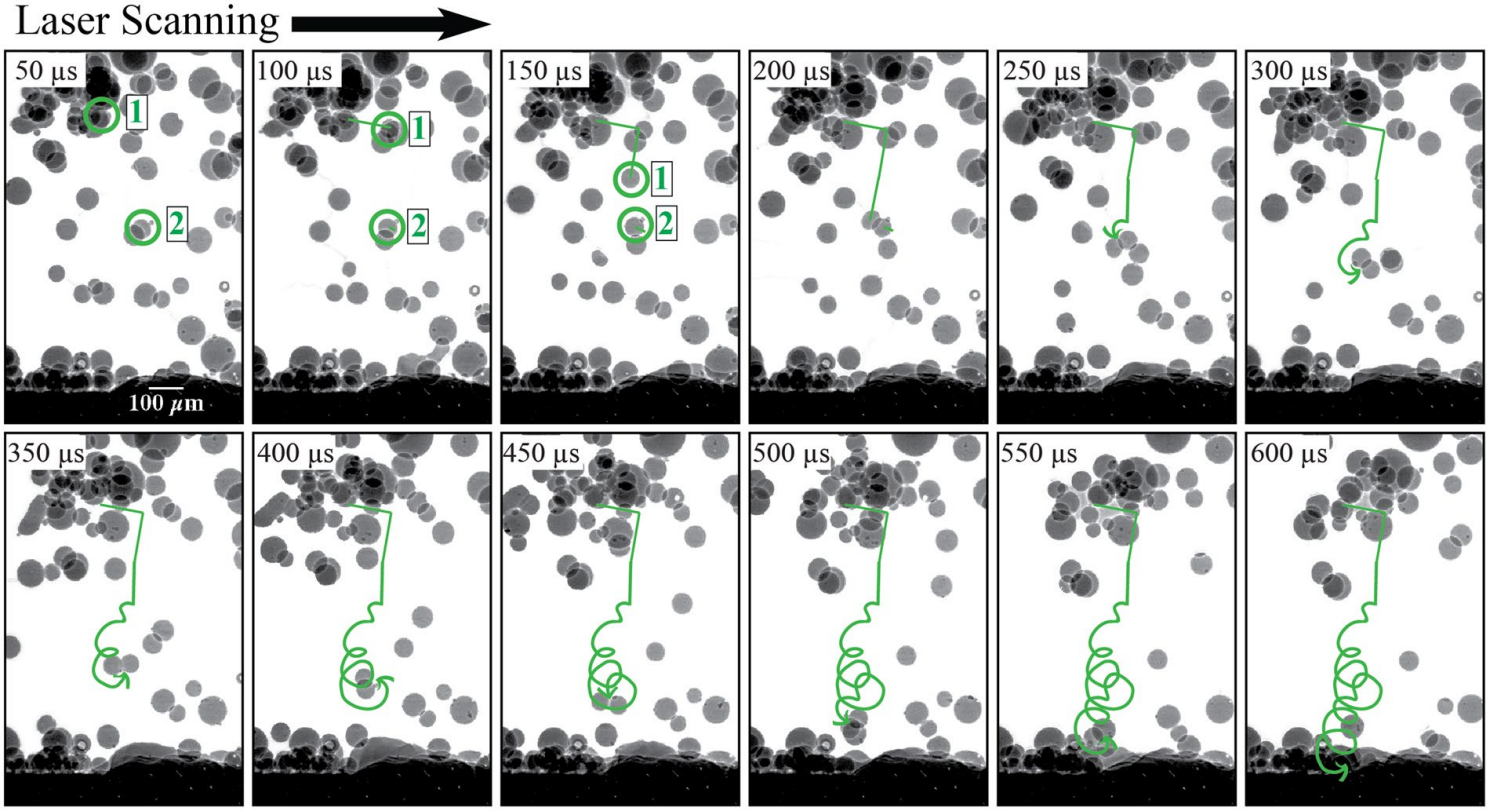
Interaction of two particles that rotate into the melt pool during a keyhole mode experiment where laser power is 250 W and the scan speed is 300 mm/s.

Though most particles that flow vertically toward the melt pool scatter due to the vapor-plasma plume at the substrate surface, high-speed x-ray imaging also captures individual particles that enter the melt pool. Interaction among flowing particles introduces additional force for particles to penetrate the vapor plume and enter the melt pool. As seen in Fig. 6, the laser beam scans toward the right and leads to the scattering of particles at 50 *μ*s. From 50 to 200 *μ*s, the particle farther away from the underlying melt pool (circled in green and denoted by “1”) collides with another particle (also circled in green and denoted by “2”). At 200 *μ*s, the surrounding vapor-plasma plume heats both circled particles to fuse them at their boundaries. These particles are sintered together and begin to propel with rotation into the melt pool from 200 *μ*s to 600 *μ*s, when the coalesced particles enter the melt pool. Increased mass leads to increased momentum, allowing the particles to penetrate the vapor-plasma plume barrier. The sintered particles move at a velocity about 1 m/s, or close to the threshold velocity of about 1 m/s for particles to enter the melt pool. Increased mass and the momentum that initiate with the coalescence of two particles lead to a rotational motion that allows the particles to contribute mass to the DED process.

#### Particle flow due to pressure gradient

**Figure 7:**
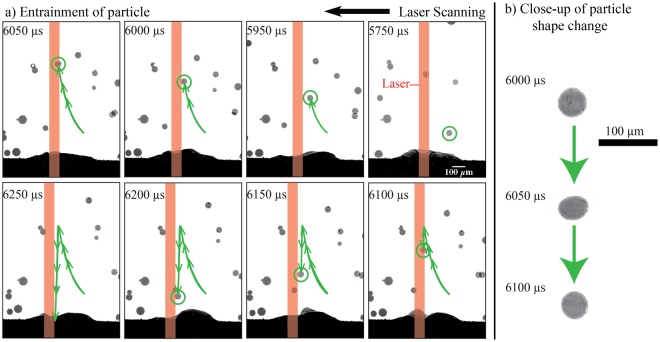
Example of vapor entrainment during a keyhole mode experiment where the laser power is 150 W and the scan speed is 100 mm/s.

The pressure gradient induced by the vapor-plasma plume at the surface of a substrate also entrains individual particles into the melt pool. Vapor driven entrainment controls nearby particles when the particle is in the path of the cavity-induced pressure gradient as seen in Fig. 7. In Fig. 7a, the laser scans from right to left. From 5750 to 6000 *μ*s, the circled particle is at a great enough distance from the melt pool that the vapor-plasma plume scatters the particle away from the substrate surface with no indication of rotation. At 6050 *μ*s, the particle makes contact with the laser beam and changes shape, indicating either rotation, melting, or both, as shown in Fig. 7b. The laser beam also vaporizes a section of particle surface, creating a localized metal vapor plume. The localized vapor plume causes a jet-like pressure gradient at the particle surface, pushing the particle toward the melt pool with a velocity of about 4 m/s. At such an elevated velocity, the particle penetrates the vapor-plasma plume without the aid of a carrier gas. This shows that localized vapor driven entrainment can capture a particle at least 200 *μ*m above the surface of the melt pool.

**Figure 8:**
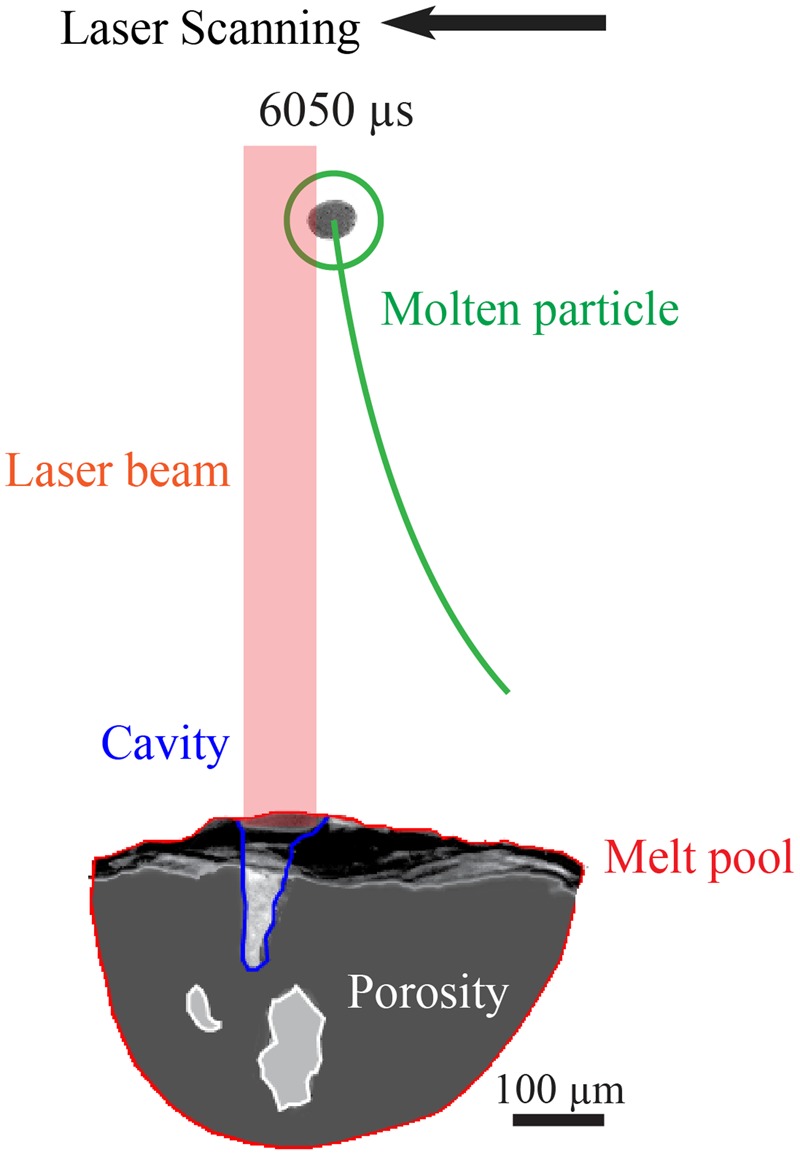
Example of vapor-driven entrainment of a single particle.

The molten particle in Fig. 7b at 6050 *μ*s also attenuates the laser beam path. Figure 8 illustrates additional evidence of laser attenuation at the 6050 *μ*s frame from Fig. 7. Figure 8 shows a segmented representation of the trajectory of the molten particle relative to the underlying melt pool. The light-intensity areas within the melt pool include the laser-induced cavity and porosity. The cavity occurs where the substrate material is vaporized by the laser, generating a vapor-plasma plume. Compared to above the substrate surface, the cavity generates the greatest velocities of the vapor-plasma plume. With the keyhole mode of laser-matter processing, the laser-induced cavity extends to the bottom of the melt pool. However, when the laser is suddenly obstructed or turned off, the penetration of the cavity into the substrate simultaneously collapses and decreases in depth. This collapse causes rapid shrinkage and a recoil pressure within the melt pool, inducing two large keyhole pores below the cavity that are around 100 *μ*m in size.

The decrease in the cavity penetration depth into the melt pool indicates loss of absorptivity of the laser into the substrate surface, causing sudden changes in the thermal history of the melt pool and of the additive manufactured build. Figure 7a shows that at 6100 *μ*s, the trajectory of the molten particle diverts downwards towards the melt pool. As the particle regains its shape and size during its downward trajectory toward the melt pool, the laser beam path no longer attenuates. The laser-induced cavity within the melt pool regains its full depth and coalesces with the large keyhole pores. This fluctuation in cavity depth and melt pool shrinkage reveal the fluctuations with the absorptivity of the laser into the substrate surface as a particle interacts with the laser beam.

#### Particle flow due to both pressure gradient and interaction with other particles

**Figure 9:**
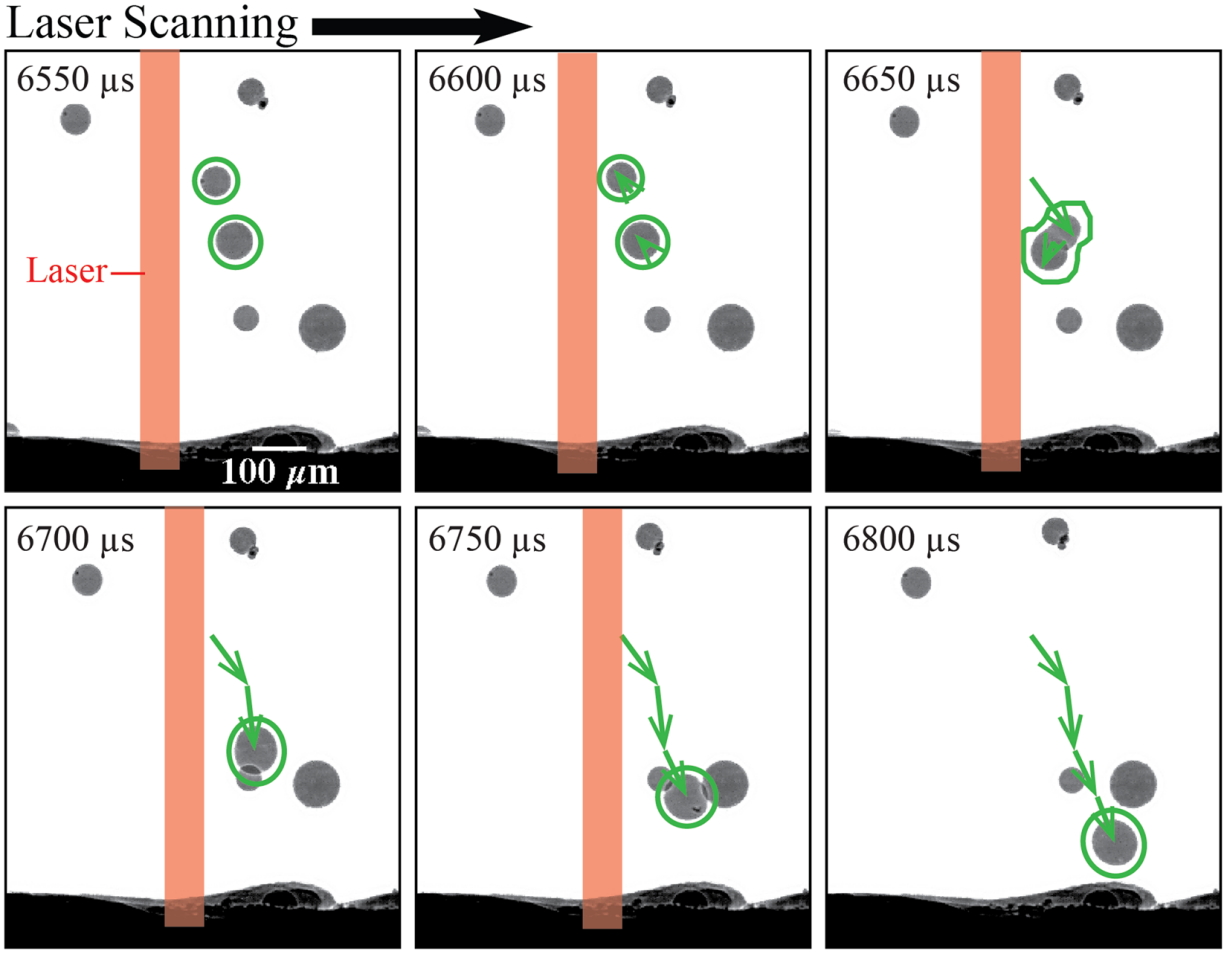
Example of two particles melting into one as they enter the melt pool during a conduction mode experiment where laser power is 150 W and the scan speed is 500 mm/s.

Figure 9 shows a conduction mode experiment where both a localized pressure gradient and particle leads to mass addition into the melt pool. As the laser scans toward the right, the vapor-plasma plume from the substrate surface entrains the two circled particles upward from 6550 to 6600 *μ*s. At about 6600 *μ*s, the surface of a smaller particle makes contact with the laser beam, causing a localized vapor-induced pressure gradient at the particle surface. The smaller particle also melts into a liquid particle during this case of laser attenuation.

As seen at 6650 *μ*s, the molten smaller particle rapidly moves downward, making contact with the larger circled particle. In this case, the temperature of these two particles was sufficiently high to cause fusing at the boundary and subsequent melting into a single larger particle, as seen from 6650 and 6700 *μ*s. The coalescence into a single particle can be attributed to a number of possible factors, including the fact that the smaller particle was at a molten state when it made contact with the larger particle, conducting the larger particle to its melting point. Other possible factors for a full melt of both particles include the proximity of both particles to the laser beam at 6650 *μ*s and the possibility that the larger particle also makes contact with laser beam before 6550 *μ*s. The momentum induced as the two particles coalesce does not create a rotational propelling motion into the melt pool as seen in Fig. 6, but mostly a linear trajectory downwards toward the melt pool. Even as the laser is turned off at 6800 *μ*s in Fig. 9, the coalesced particle is still in its trajectory toward the solidifying substrate surface. Because the cooling behavior of the melt pool in conduction mode experiments is more rapid than in keyhole mode experiments, the coalesced particle only partially melts into the substrate.

#### Particle behavior summary

Overall, the various mechanisms of particle flow behavior depend on the laser beam path and can attenuate the laser. The laser-induced vapor-plasma plume scatters and entrains particles away from the melt pool. Entrainment away from the melt pool can lead particles to interact with each other or make contact with the laser beam path. These interactions induce a momentum or pressure gradient that allow for particles to enter the melt pool. In turn, as particles flow into the path of the laser beam, the resulting melt pool fluctuates due to fluctuating attenuation. Two instances of vapor entrainment of the particle occur, where the first, more global vapor-induced pressure gradient scatters the particle upwards in the direction of the growing vapor-plasma plume. The second instance of vapor entrainment occurs when the particle makes contact with the laser beam, vaporizing a fraction of the particle surface. The localized vapor jet pushes the particle into the melt pool.

### Influence of two laser beam passes on porosity and cooling behavior

With two-pass experiments, cooling behavior is observed after laser processing on both room temperature and heated substrates. During rapid cooling in DED, porosity traverses through the melt pool with melt pool convection velocity. Resulting porosity expands, shrinks, dissipates at the surface, or is constrained by the solidified melt pool after cooling. Scanning the laser for a second pass does not necessarily remove or change the resulting keyhole porosity from the first pass. Mass change in the melt pool can be monitored and influences the cooling rate in both passes. Trends in cooling behavior show that powder mass flow can have a significant influence in the thermal history and structure of the melt pool during the first pass.

#### Porosity evolution during solidification

Tracking the cavity geometry evolution during laser-matter interaction allows observation of the moving liquid-solid interface that surrounds the cavity zone where the laser vaporizes the substrate, as seen in Figs 4 and 5. The moving solidification front introduces more keyhole porosity formation at the liquid-solid interface. Porosity usually stirs within the melt pool, shrinks, and then dissipates. However, the melt pool boundary also acts as a constraint on keyhole pores as they are pushed out of the liquid-solid interface into the surrounding substrate.

**Figure 10:**
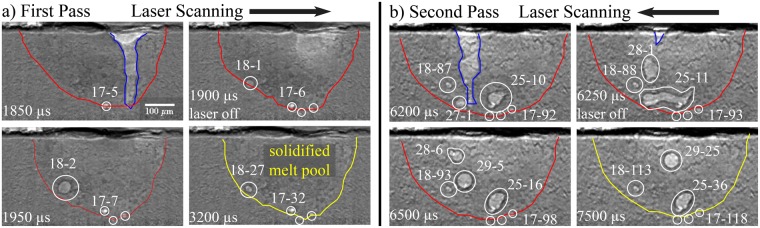
Evolution of keyhole porosity after the laser is turned off and until the substrate is solidified for two passes. The experimental conditions are 150 W laser power, 100 mm/s, 400 V voltage input into the piezo-driven powder delivery system with a pulse frequency of 100 Hz, and duty cycle of 10% for each pulse.

Porosity location, shape and size from the two-pass experiment in Figs 4 to 5 are tracked after the laser is turned off and the melt pool solidifies. A fully solidified melt pool is determined when the melt pool, and any feature within the melt pool (keyhole pores, micro-pores, and any pixel that change in intensity) become stationary. Figure 10a shows a small pore constrained by the liquid-solid interface at 1850 *μ*s (pore “17-5”), immediately before the laser turns off. As soon as the laser turns off at 1900 *μ*s, the pore disperses into three entrapped gas pores (porosity group “17-6”). In subsequent frames until the melt pool fully solidifies at 3200 *μ*s, pore group “17” oscillates in size from 300 to 340 *μ*m^2^ until it reaches an equilibrium at about 320 *μ*m^2^. As soon as the laser turns off in 1900 *μ*s, shrinkage causes a keyhole pore, porosity group “18”, within the melt pool. At 1950 *μ*s, the keyhole pore (“18-2”) expands from 470 to 1213 *μ*m^2^ as the solidifying melt pool still stirs and shrinks. In subsequent frames, porosity group “18” decreases in size and maintains equilibrium when the substrate fully solidifies at 3200 *μ*s, reaching a size of about 260 *μ*m^2^.

There is a 2.5 ms delay from the time the laser is turned off in the first pass to the beginning of the second pass, with each pass lasting 2000 *μ*s of laser illumination. During the delay, the constrained porosity from the first pass, groups “17” and “18”, remain stationary. During the second pass, these constrained porosity groups from the first pass remain within the melt pool and appear in the final solidified part after the second pass. Eight groups of pores form and dissipate into the melt pool during the second pass, resulting in two large keyhole pores, groups “25” and “27”, at 6200 *μ*s, immediately before the laser turns off. When the laser turns off at 6250 *μ*s, the two large pores at 6200 *μ*s coalesce and another keyhole pore forms closer to the surface as a result of rapid shrinkage. After 250 *μ*s, at 6500 *μ*s, the coalesced pore splits into two pores (“25-16” and “29-5”). The large keyhole pore from 6250 *μ*s, “28-1”, shrinks and moves vertically toward the surface, as seen at 6500 *μ*s with porosity group “28-6”. Until the build solidifies at 3000 *μ*s, pore “28” dissipates at the substrate surface, “29” epitaxially travels toward the surface and slightly decreases in size, and “20” is constrained by the liquid-solid interface. In addition to the porosity that remain from the first pass, “17” and “18”, two large keyhole pores of almost 100 *μ*m in size result after the part solidifies after two passes. The capability to monitor the evolution of porosity location, shape and size during and after a build can reveal the mechanisms behind defects in additive manufactured parts, as small fluctuations to the laser power, scan speed and overall absorptivity influence the solidified structure.

#### Cooling behavior with mass change

**Figure 11:**
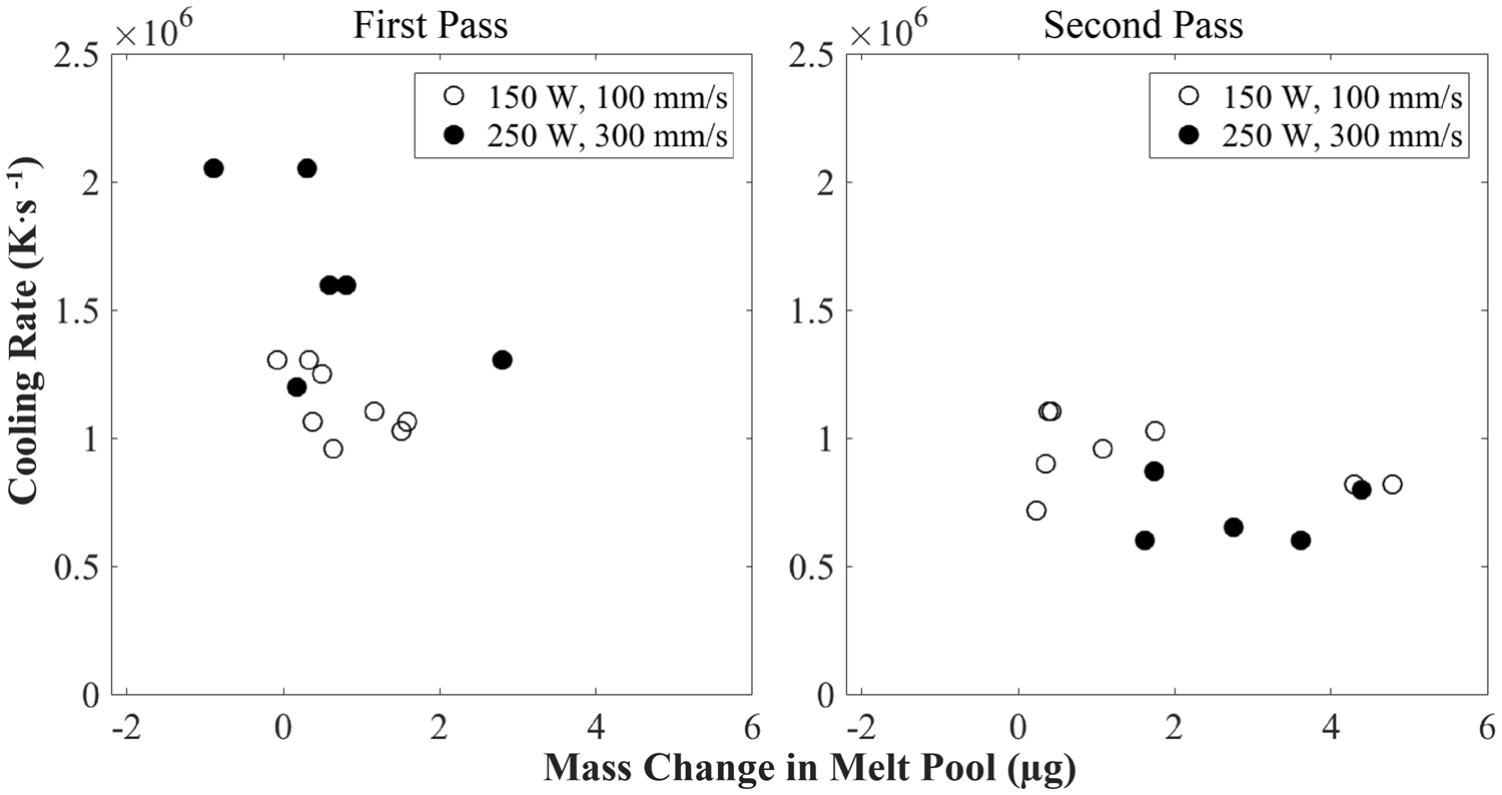
Cooling rate with the change of mass in the melt pool for each pass during each keyhole mode experiment. Each data point represents a pass in one experiment.

Mass changes in the melt pool influence the dynamics of the melt pool, its cooling behavior and hence, the structure and properties of the final part. Mass change in the melt pool is monitored and calculated by tracking the size of any particle that enters or ejects out of the melt pool, counting addition as positive mass change and spatter as negative mass change. Mass subtraction does not take evaporation into account. The cooling rate is determined by using the difference between the extreme temperatures of the liquid phase for Ti-6Al-4V. More specifically, the temperature change is the difference between the vapor/liquid and liquid/solid temperatures (3315 K and 1928 K, respectively)^40^. Time change is determined by counting the number of frames it takes for the surface of the melt pool above the substrate surface to solidify and remain stationary after the laser turns off. The resulting cooling rate is compared with the total mass change of each pass in each keyhole experiment, as seen in Fig. 11.

The trends in cooling for both sets of keyhole experiments match what previous modeling studies predict^20,26,41^. The cooling rates in the first pass are greater than those in the second pass. At the beginning of the first pass, the laser heats up a substrate with an initial state at ambient temperature, resulting in a large thermal gradient. When the second pass begins, the substrate is still hot from the first pass and the thermal gradient after the pass is not as significant. The difference in cooling rates between the first and second passes are greater for the 250 W, 300 mm/s set of experiments than that of the 150 W, 100 mm/s set of experiments because the more powerful laser causes a greater thermal gradient during the first pass, but also results in a hotter substrate when the second pass begins.

As the amount of mass in the melt pool increases, the cooling rate generally decreases as more time is required to melt and cool a greater amount of material and a larger melt pool. The dependence on mass change is greatest during the first pass because more time is required to melt additional material, or particles, in ambient temperature and result in rapid cooling whereas the initial elevated temperature at the beginning of the second pass overcomes the influence of mass change at ambient temperature. The greater scan speed in the 250 W, 300 mm/s experiments as compared to the 150 W, 100 mm/s experiments results in a greater spread in cooling rates and increased dependence on mass during the first pass.

With a laser beam size of 80 *μ*m, the sensitivity of laser absorptivity and cooling to mass change is greater than that of commercial additive manufacturing systems that use laser beam sizes of up to 5 mm. This study reveals a small-scale representative study of the influence of mass change in the melt pool on the cooling behavior of a build. Cooling behavior can be attributed to process parameters, powder flow, porosity formation, porosity evolution, and mass change into or out of the melt pool.

## Conclusions

The scientific purpose of these *in-situ*, high-speed, X-ray imaging experiments is to capture and quantify physical phenomena during DED processes for controlled processing and materials development, which are still relatively unclear in the scientific community. High-speed X-ray images that reveal the laser-matter interactions in various modes of DED processing can aid in the validation of thermal, thermo-fluid dynamic and thermo-mechanical models. In the case of gravity-fed, low powder mass flow, the laser-induced vapor plume scatters particles away from the melt pool with velocities of up to 10 m/s. Surface tension of the melt pool entrains particles on the surface to enter the melt pool or ejects hot particles. Various mechanisms allow for individual in-flight particles to enter the melt pool, including particle interactions and localized pressure gradients from a scanning laser. Particles that flow into the melt pool influence the stability of the laser-induced cavity and the surrounding melt pool. Porosity continually forms, stirs within the melt pool, shrinks into the liquid phase of the substrate, or is constrained at the solidification boundary. Changes in mass entering or ejecting out of the melt pool influence the resulting cooling rates of the melt pool, which can reach up to 10^6^ K/s.

Currently, IR sensors can only capture qualitative temperature data during the build due to the dynamic temperature and phase changes during the process. Most IR sensors do not have a high enough temporal resolution to capture the rapid cooling behavior of DED processes. These cooling rates have shown to influence unique phase transformations, defect morphologies, and grain structures. This study elucidates possible causes of porosity. Further studies are necessary to understand interesting mechanisms of particle entrainment, particularly of particles that attenuate the laser beam path. Controlling individual particle trajectories relative to a laser beam can lead to more conclusive observations about how particles enter the melt pool. This work reveals the necessity of an inert carrier gas to aid particle flow. Without carrier gas, most particles scatter away from the melt pool, whereas carrier gas allows particles to penetrate the laser-induced vapor-plasma plume. Future work that investigates the influence of carrier gas pressure and velocity is required to capture the phenomena in more representative DED processing. Coupling high-resolution thermal monitoring can also aid in further understanding of cooling and more specifically, solidification behavior of the melt pool.

## Methods

The Ti-6Al-4V powders deposited onto the substrate surface were recycled Advanced Powders and Coatings Inc. plasma-atomized powders of 45 to 106 *μ*m in diameter. The substrate materials were titanium alloy Grade 5 sheet metal plates from McMaster Carr and the same material used in^33^. The vacuum chamber, laser system, high-speed synchrotron X-ray imaging system, and image analysis using the second derivative of intensity profiles of each image from past work in the beamline^33^ were also used in this work. The custom-made stainless steel vacuum chamber was filled with argon gas for an inert environment. The laser system was an IPG YLR-500-AC ytterbium fiber laser. The galvanometer scanner, intelliSCANde30 scanner with a digital encoder from SCANLAB GmbH, Puchheim, Germany, was used to position the laser beam.

## Data Availability

The raw and processed data are available from the corresponding authors on reasonable request.
